# Visionary Leadership in Cancer Care: An Interview With Professor Timothy J. Eberlein

**DOI:** 10.1002/ags3.70211

**Published:** 2026-03-07

**Authors:** Ken Shirabe, Koshi Mimori, Masaki Mori, Yuko Kitagawa

**Affiliations:** ^1^ Department of General Surgical Science Graduate School of Medicine Maebashi Gunma Japan; ^2^ Department of Surgery Kyushu University Beppu Hospital Beppu Oita Japan; ^3^ Department of Gastroenterological Surgery Tokai University School of Medicine Isehara Kanagawa Japan; ^4^ Department of Surgery Keio University School of Medicine Tokyo Japan

**Keywords:** academic medicine (virtuous cycle), cancer center leadership, surgical education and mentorship

## Abstract

This is a short interview with Professor Timothy J. Eberlein, conducted by Professor Ken Shirabe, President of the Japanese Society of Gastroenterological Surgery, explores the vision, strategies, and leadership behind developing the Siteman Cancer Center into a world‐class institution. Professor Eberlein emphasizes two central goals: becoming experts in every type of cancer and ensuring that every patient feels uniquely cared for. He highlights the importance of large‐scale clinical programs in sustaining a virtuous cycle of academic medicine, where clinical revenue supports research and education, and innovations attract both patients and trainees. A distinctive element of his philosophy is individualized training, offering flexibility for surgical residents to focus on areas such as oncology, minimally invasive surgery, or education. He underscores the critical role of mentorship, collaboration, and laboratory organization in nurturing surgeon‐scientists. Balancing surgery and research is described as a delicate challenge, but one that can be overcome with proper mentorship and collaboration. Leadership is about leading by example, solving problems for others, and respecting every member of the healthcare team. He stresses that surgery is the ultimate team sport, with outcomes dependent on the collective contributions of diverse professionals. Finally, his message to the next generation of surgeons and oncologists in Japan is to maintain their passion, embrace innovation, and realize that the future holds opportunities, such as genetic therapies, vaccines, and immune therapies that will revolutionize cancer treatment when combined with surgical expertise.

## Graphical abstract

Professor Timothy J. Eberlein and Professor Ken Shirabe, President of the Japanese Society of Gastroenterological Surgery, during their interview discussing the vision, strategic framework, and leadership that shaped the development of the Siteman Cancer Center into a world‐class institution.

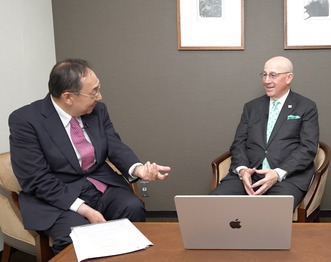

## 1 Opening and Introduction

We disclose the third session of the special series, “Interview with World Class Authorities” presented by *Annals of Gastroenterological Surgery* to invite renowned leaders in surgery from around the world for a short interview. We welcome Professor Timothy Eberlein from Washington University School of Medicine, a truly distinguished figure in American surgery. Professor Ken Shirabe, President of the Japanese Society of Gastroenterological Surgery is the interviewer.

### 1.1 Building a World‐Class Cancer Center

**FIGURE 1:**
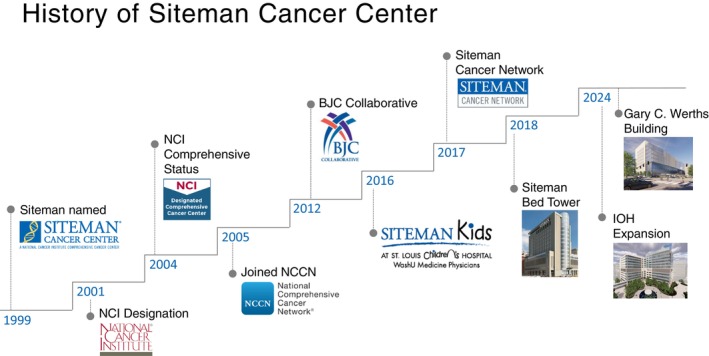
Historical milestones and institutional development of the Siteman Cancer Center. This timeline illustrates the major milestones in the evolution of the Siteman Cancer Center from its naming in 1999 through recent expansions. Key developments include National Cancer Institute (NCI) designation in 2001, attainment of NCI Comprehensive Cancer Center status in 2004, and joining the National Comprehensive Cancer Network (NCCN) in 2005. Subsequent institutional growth involved the establishment of collaborative frameworks such as the BJC Collaborative, expansion of the Siteman Cancer Network, construction of the Siteman Bed Tower, and major infrastructure developments including the Gary C. Werths Building and the Institute of Oncology and Hematology (IOH) expansion completed in 2024, reflecting sustained growth in clinical, research, and network capacity.

Ken Shirabe (KS) First of all, I would like to talk about building a world‐class cancer center. I read this figure and was so impressed (Figure 1). It is an outstanding success. But I think mission and vision are very important for each cancer center. I was impressed that you paid very special attention to the community.

Timothy J. Eberlein (TJE) Washington University did not have a cancer center designated by the National Cancer Institute. They had tried for many years. When I was recruited as Chair of Surgery, I was asked to establish a new cancer center with the hope of NCI designation (Figure 1). We set two major goals. First, to be experts in every kind of cancer—colon, blood, brain, bone, etc. Second, to make every patient feel they were the only patient we cared for. Our mission was to prevent cancer in the community and transform patient care through scientific discovery, building on the great science of Washington University. Our vision was to be world‐class leaders in cancer care, but one patient at a time.

KS Yes. This is so impressive, this phrase. Is there a special meaning behind this sentence?

TJE What really made it important is we wanted every patient to feel they were the most important patient that came to our center, and that every patient, regardless of their background, would be able to access the paradigm‐shifting treatments that we were developing.

KS Wow, that is great! I think that this slide shows the minority and the medically un‐serviced. The percentage of these showing here is very common in the United States?

TJE One of the unique features of our cancer center is we serve a very large area in the center of the United States. Within that geography are patients who are underserved, who have difficulty accessing medical care. That becomes a special challenge for us. The other thing is that patients tend to have a large number of cigarette smokers, they are obese, they do not have good diet and exercise, and so they also have a high propensity for getting access to heavy metals. Many people worked in the mining industry doing copper, lead, and other types of mines, with industrial exposure. For all those reasons, it is a very challenged community of people who require cancer care. One of the commitments that we made is that we wanted to be a leader in accrual to patient clinical trials. All advances in cancer are made through clinical trials. We wanted to include underserved minority and rural patients. We now run between the third and fourth most number of clinical trial accruals in the United States.

KS I was so impressed. Everyone can access your cancer center in Washington and the whole world.

TJE Yes. We actually have patients from all 50 states in the United States who come to Siteman [1].

KS Yes. But underserved patients?

TJE And underserved, yes, and rural. Every quarter, the senior leadership of the cancer center reviews all clinical trials and accrual to assure we are including underserved, rural, and minority patients [2].

KS That is great. But maybe a little bit difficult in the point of budget.

TJE We have grown very large—we are the third‐largest cancer center in the United States. That scale allows us to afford to take care of underserved patients [3]. Last year, we provided $159 million of free care. But you have to be very large to afford that. It is part of our mission: to be a safety net provider. Not every cancer center does that.

KS Yes, I think so. It was maybe because of the strong vision of the leader.

TJE We have grown dramatically over the years. Our research budget is nearly $200 million a year in research grants and studies. Innovations attract patients because they realize they can get something unique at our center.

KS That is incredible. Everyone can access your center in Washington and also worldwide.

TJE Yes. We have wonderful providers—surgeons, oncologists, researchers, nurses, and thousands of people. This year we will care for over 75 000 unique patients.

KS Recently, the government of the United States is changing their policy, and Diversity/Equity/Inclusion (DEI) may be a little difficult [4]. How do you think about that?

TJE It is a challenge. Each administration changes directives. Under President Trump, many directives changed. We adhere to all those directives but still maintain our mission of access for anyone. We adapt while following the law.

KS I have heard many companies have withdrawn support to DEI.

TJE Yes, and just like, Japan has negotiations on tariffs and trade, we must also adjust. But our programs are about access for patients of all backgrounds. We had no preference for any one type of patient.

### 1.2 SPORE Grants and Research Support

**FIGURE 2:**
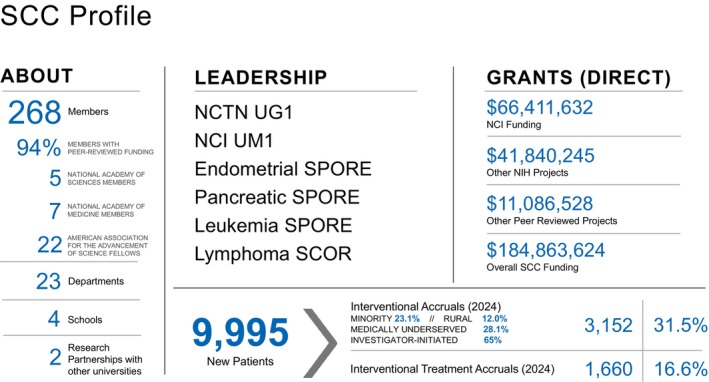
Institutional profile and research–clinical capacity of the Siteman Cancer Center. This figure summarizes the comprehensive profile of the Siteman Cancer Center (SCC), highlighting its research funding portfolio, clinical trial activity, and organizational scale. Total peer‐reviewed research funding exceeds $184 million, including $41.8 million in National Cancer Institute (NCI) funding and $66.4 million in direct grant support. SCC comprises 268 members across 23 departments and four affiliated schools, with 94% of members supported by peer‐reviewed funding. In 2024, SCC enrolled 9995 new patients and demonstrated robust interventional trial accruals, including high proportions of investigator‐initiated studies (65%) and participation from minority (23.1%), rural (12.0%), and medically underserved populations (28.1%). The center also maintains strong national leadership through multiple NCI mechanisms (UG1, UM1, SPOREs, and SCORs), National Academy memberships, and active research partnerships with external universities.

TJE The NCI has Specialized Programs of Oncologic Research Excellence (SPORE). Siteman has three—endometrial, pancreatic, and leukemia (Figure 2). We also have a similar SCORE grant in lymphoma. Interestingly, two of these SPOREs are led by surgeons.

TJE So far, we have managed to use philanthropy funds—donations targeted to new research initiatives and supporting clinical trials. These allow us to continue despite budgetary changes.

### 1.3 The Virtuous Cycle of Academic Medicine

**FIGURE 3:**
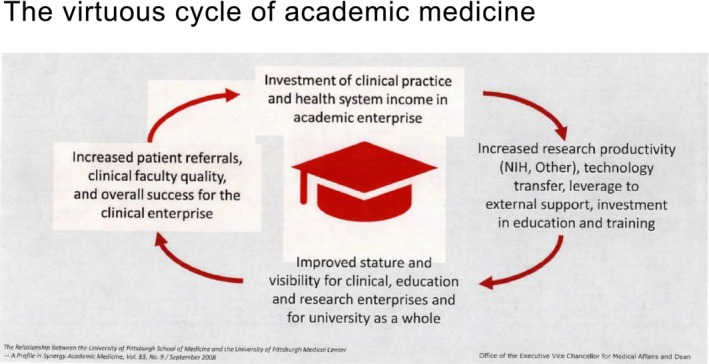
The virtuous cycle of academic medicine. This schematic illustrates the virtuous cycle underlying academic medicine, in which clinical care, research, and education mutually reinforce one another. High‐quality patient care generates clinically relevant questions that drive innovative research, while research discoveries are translated back into improved diagnostics, therapeutics, and clinical outcomes. These advances, in turn, enhance educational excellence by training the next generation of clinicians and scientists within an environment of inquiry and discovery. Sustained integration of these elements strengthens institutional impact, attracts talent and funding, and ultimately promotes continuous advancement in patient‐centered academic medicine.

KS I have read your paper, “The Changing Paradigm of Surgical Education.” [5] Very impressive. You are a researcher and a teacher. Could you explain the virtuous cycle of academic medicine (Figure 3)?

TJE To support research and education, you need revenue from a large clinical program. That income fuels research and education. Innovation then attracts trainees and patients, which sustains the cycle. It is a virtuous cycle of academic medicine. It is not only just the cancer center but also the Department of Surgery. Our department is routinely first or second in research funding in the United States. We built innovative educational programs, which attract top trainees. That combination requires large clinical programs to support the costs.

KS I found that “personalized” seems to be an important keyword for you—education and treatment.

TJE I would say “individualized.” Trainees want different paths. Some want to be world‐class clinicians. Some focus on outcomes, safety, and quality measures. Some produce innovative clinical trials. Some want to be educators, developing new training paradigms [6]. We developed a unique “Flexibility in Surgical Training” program, allowing trainees to dedicate a third of their last 3 years to a specialty. Many residents graduate with more operative experience than fellows elsewhere. We still have surgeon scientists [7]—though fewer than 1000 American surgeons have NIH grants. We help by providing financial support, protected time, strong mentors, critical mass in laboratories, and collaborations. Collaboration is crucial—showing feasibility through teamwork rather than doing all work alone.

### 1.4 Balancing Surgery and Research

KS Recently in Japan, residents are too busy to do research. How do you think about that?

TJE It is a delicate balance. Like being a baseball player also trying to play pro hockey. Very few can do both. That is what surgeons face—balancing surgery and research. Mentorship and collaboration help. Surgeons are smart and hardworking but need to learn the rules. With guidance, they succeed. Surgeons tend to look at the big picture: patients with complex issues beyond the operation itself. In research, surgeons identify gaps that improve outcomes—better techniques, systemic treatments, or combined therapies. Surgeons often excel at that perspective.

KS In Japan, provosts do not see the disease, but the patient.

TJE Yes. Trainees gather data—scans, biomarkers, genetics—but I always say, when all else fails, go talk to the patient. They will often tell you the diagnosis and treatment, even if not in technical terms. Listening to patients is vital.

### 1.5 Leadership and Mentorship

TJE Leadership evolves from being a successful physician and surgeon. I always tried to hire people smarter than me and lead by example. Today, leadership also requires formal training. Many trainees attend business school to learn leadership skills. I learned from Dr. Henry Bahnson, a brilliant cardiothoracic surgeon. At first, I thought leadership meant being at the top of the pyramid. As chair, I realized the pyramid was inverted—I was there to solve others' problems. Leadership is about helping others implish their goals.

### 1.6 Surgeons in Team‐Based Medicine

KS What is your policy about the role of surgeons in team‐based medicine?

TJE Surgery is the ultimate team sport. A surgeon is only as good as the anesthetist, intensivist, perfusionist, nurse, etc. If you do not respect your team, you would not have good outcomes. I tell trainees: know everyone in your hospital—even transporters, lab staff, blood bank workers. They are critical. Respecting them makes the institution function and builds strong teams.

### 1.7 Message to the Next Generation

TJE We are in an incredible time. Gastrointestinal cancers are many diseases, and we are understanding their biology better. Maintain the passion you had when you first entered the operating room. The next generation will accomplish things we only dreamed of—genetic therapies, vaccines, immune therapies combined with surgical skills. That is the promise of the future.

## 2 Concluding Remarks

In conclusion, Professor Eberline's insights provide not only a roadmap for building a world‐class cancer center but also timeless lessons in leadership, mentorship, and patient‐centered care. His reflections emphasize that innovation, inclusivity, and passion will continue to shape the next generation of surgeons and oncologists, ensuring meaningful progress in the fight against cancer for years to come.

## Author Contributions

**Ken Shirabe:** interviewer, supervision. **Koshi Mimori:** writing – original draft. **Masaki Mori:** project administration. **Yuko Kitagawa:** project administration.

## Funding

The authors have nothing to report.

## Conflicts of Interest

The authors declare no conflicts of interest.
